# Macrophage plasticity in thyroid eye disease: dual effects of inflammation and fibrosis

**DOI:** 10.3389/fimmu.2026.1782204

**Published:** 2026-03-04

**Authors:** Xinyu Li, Zhangjun Ren, Lanlan Gao, Chao Xiong, Hongfei Liao

**Affiliations:** 1 School of Optometry, Jiangxi Medical College, Nanchang University, Nanchang, China; 2 Jiangxi Research Institute of Ophthalmology and Visual Science, Nanchang, China; 3 Jiangxi Provincial Key Laboratory for Ophthalmology, Nanchang, China; 4 National Clinical Research Center for Ocular Diseases Jiangxi Province Division, Nanchang, China; 5 The Affiliated Eye Hospital, Jiangxi Medical College, Nanchang University, Nanchang, China; 6 Jiangxi Clinical Research Center for Ophthalmic Disease, Nanchang, China

**Keywords:** autoimmune disease, fibrosis, functional status continuum, Graves’ ophthalmopathy, inflammation, macrophages, thyroid eye disease

## Abstract

Thyroid Eye Disease (TED), also known as Graves’ ophthalmopathy, is an organ-specific inflammatory disorder associated with autoimmune thyroid dysfunction. Its primary pathological features include immune cell infiltration of orbital tissues, fat hyperplasia, and fibrotic remodeling. The pathogenesis centers on abnormal expression of TSHR on orbital fibroblasts and immune attacks mediated by autoantibodies. Recent studies increasingly reveal that infiltrating immune cells, particularly highly plastic macrophages, do not simply divide into static M1/M2 phenotypes. Instead, they exist within a functional continuum precisely regulated by transcriptional and epigenetic mechanisms, dynamically adjusting their functional states in response to microenvironmental signals. Along this continuum, macrophages in early disease stages lean toward the pro-inflammatory pole. Activation of transcription pathways like NF-κB, coupled with concomitant epigenetic remodeling, drives the release of inflammatory mediators such as IL-6 and TNF-α, thereby initiating and amplifying inflammatory cascades. During disease progression, macrophages shift toward the pro-fibrotic end. Their functional state is influenced by the sustained activation of transcriptional programs like TGF-β/Smad and STAT3, as well as the consolidation effects of epigenetic mechanisms such as DNA methylation and histone modifications. This facilitates pathological tissue repair and fibrosis through signaling pathways including GAS6-AXL and PDGF. This review systematically examines the dynamic regulatory role of macrophages in TED, delves into their complex interaction networks with fibroblasts, adipocytes, and lymphocytes. It further envisions novel therapeutic strategies targeting the macrophage functional continuum and its underlying transcriptional and epigenetic regulatory mechanisms. This aims to establish a pathological framework for TED centered on the spatiotemporal evolution of macrophages, providing theoretical foundations and translational perspectives for developing temporal and precision therapies that transcend conventional immunosuppression.

## 1 Introduction

Thyroid Eye Disease (TED) is the most representative extra-thyroid manifestation of Graves’ disease (1–3). The pathological process is divided into two distinct phases: an active phase driven by immune inflammation and a quiescent phase characterized by tissue remodeling (4, 5). Currently, first-line treatments such as systemic glucocorticoids and immunosuppressants can effectively control active inflammation, but they have limited efficacy against established fibrotic lesions during the quiescent phase (6–8). This therapeutic impasse underscores the urgent need to reexamine the underlying mechanisms driving disease stage transitions.

The essential characteristic of the monocyte-macrophage lineage lies in its remarkable diversity and plasticity. In response to signals such as IFN-γ, Toll-like receptor agonists, or IL-4/IL-13, macrophages can respectively enter classical activation (M1) or alternative activation (M2) states. However, these two phenotypes are not discrete functional endpoints but rather occupy opposite ends of a continuous activation spectrum, the actual functional state of macrophages is always distributed along a continuum precisely shaped by microenvironmental signals (9–11). The construction of the macrophage functional continuum is rooted in multi-level molecular regulation. At the transcriptional level, members of the PPAR, KLF, IRF, STAT, NF-κB, and HIF families form an intersecting regulatory network, whose combined expression patterns directly define the spatial position of cells along the activation spectrum. At the epigenetic level, histone methylation and acetylation modifications exert “bias regulation” on the accessibility of these transcription factors by altering chromatin accessibility—conferring transient plasticity to macrophage signaling responses while leaving enduring imprints of functional states. Thus, M1 and M2 are no longer fixed phenotypes but observable endpoints of this dynamic continuum under specific signaling combinations (12–14). Single-cell RNA sequencing indicates that macrophage subpopulations form a continuous spectrum rather than discrete clusters in both mouse and human tissues (15). Building upon this advancement, this review proposes the following core hypothesis: the functional continuum of macrophages, alongside their concomitant immunometabolic reprogramming and dynamic transcriptional and epigenetic regulation, constitutes the central regulatory mechanism driving the progression of TED from active inflammation to quiescent fibrosis.

**Figure 1:**
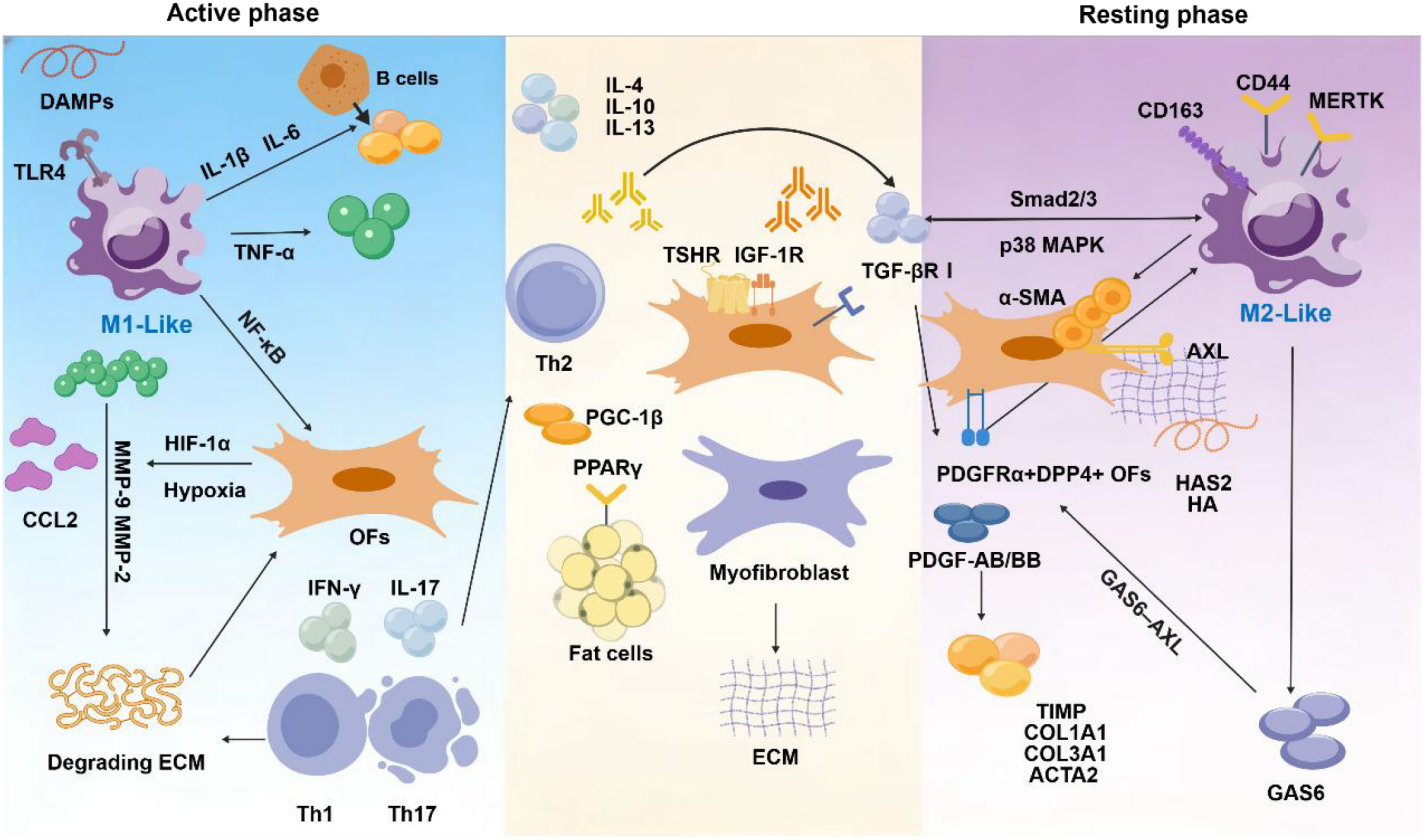
Macrophage plasticity in thyroid eye disease: dual effects of inflammation and fibrosis.

This review aims to transcend the classical M1/M2 binary paradigm by establishing an integrated framework that systematically elucidates the dynamic role of macrophages in the pathological progression of TED for the first time. We will focus on analyzing how macrophages respond to microenvironmental signals at different disease stages, completing a functional transition from a pro-inflammatory state to a pro-fibrotic state. We will elucidate the underlying mechanisms of transcriptional reprogramming, epigenetic modifications, and metabolic restructuring driving this transition. Furthermore, we will reveal how macrophages form positive feedback loops with orbital fibroblasts, adipocytes, and T/B lymphocytes, collectively propelling disease progression. (**Figure 1**) study.

Macrophage Plasticity in Thyroid Eye Disease. Schematic diagram illustrating the phenotypic continuum of macrophages during the TED active phase (left) and resting phase (right). During the active phase, DAMPs and hypoxia signals (HIF-1α) induce M1-like polarization via the TLR4 pathway, accompanied by Th1/Th17 and B-cell activation. This leads to secretion of pro-inflammatory factors, CCL2, and MMP-2/9, degrading the ECM and activating orbital fibroblasts (OFs). concurrently, Th2-derived IL-4/IL-13 and PPARγ signaling drive OF adipogenic differentiation. During the quiescent phase, PDGFRα^+^DPP4^+^ OFs respond to PDGF-AB/BB and GAS6, upregulating fibrotic genes (α-SMA, COL1A1, etc.) via TGF-βR/Smad2/3 and p38 MAPK pathways to promote ECM deposition. Macrophages undergo a shift towards an M2-like phenotype (CD163^+^, MERTK^+^, CD44^+^), further driving tissue remodeling.

## 2 Complexity and diversity of macrophage polarization profiles: their dynamic role in TED pathogenesis

Macrophages, as core effectors of the innate immune system, play multifaceted roles in immune defense, inflammation regulation, homeostasis maintenance, and tissue repair. Their functions extend far beyond those of simple effector cells, demonstrating remarkable plasticity and functional heterogeneity (16–19). Its function is not predetermined, but rather a dynamic output resulting from the integration of local microenvironmental signals by the gene expression program (20, 21). Research indicates that macrophages constitute the second largest proportion of immune cells within the orbital adipose tissue (OAT), and their abundance is significantly higher in TED patients compared to normal controls (13). Traditionally, to understand their functional diversity, macrophages have been simplified into two static polarization states: pro-inflammatory (M1) or pro-repair/fibrotic (M2). This classification framework does provide preliminary guidance for understanding the fundamental role of macrophages in TED.

Classically activated M1-like macrophages: Typically driven by signals such as interferon-γ (IFN-γ) in combination with lipopolysaccharide (LPS) or granulocyte-macrophage colony-stimulating factor (GM-CSF) (22–24). Its polarization mechanism relies on the potent activation of the JAK-STAT1 and nuclear factor κB (NF-κB) signaling pathways (25, 26). Activated M1 cells characteristically overexpress inducible nitric oxide synthase (iNOS), catalyzing the production of large amounts of nitric oxide (NO) and secreting high levels of proinflammatory cytokines, including tumor necrosis factor-α (TNF-α), interleukin-1β (IL-1β), IL-6, IL-12, and IL-23 (27–29). Its primary function is to eliminate intracellular pathogens and strongly drive the type I helper T cell (Th1) immune response (30). However, the excessive activation and persistent presence of M1-like macrophages represent a key pathological factor that causes acute tissue injury and drives the malignant progression of chronic inflammatory diseases (31). During the TED event, macrophages infiltrating the orbital tissues predominantly exhibit an M1-like phenotype. The inflammatory storm they release, characterized by TNF-α and IL-1β, directly leads to orbital soft tissue edema, abnormal glycosaminoglycan accumulation, and early tissue damage.

Alternative-activated M2-like macrophages: This population exhibits greater heterogeneity, with polarization triggered by anti-inflammatory and reparative signals such as IL-4, IL-13, IL-10, immune complexes, or glucocorticoids. This process primarily occurs through activation of signaling pathways including STAT6, peroxisome proliferator-activated receptor gamma (PPARγ), and AMPK (31, 32). M2-like macrophages commonly overexpress arginase-1 (Arg-1), mannose receptor (CD206), and hemoglobin scavenger receptor (CD163) (33, 34). Unlike M1 cells, which metabolize arginine into NO via iNOS, M2 cells metabolize arginine into ornithine and polyamines through Arg-1, thereby promoting cell proliferation and collagen synthesis. This reflects their function-directed metabolic reprogramming (35). As TED transitions from an active phase to a quiescent phase, shifts in microenvironmental signals drive macrophages toward an M2-like phenotype, while the heterogeneity of its internal subtypes determines divergent disease outcomes:

M2a subtype: Primarily induced by IL-4 or IL-13 through the STAT6 pathway (36, 37). They highly express Arg-1, CD206, and chitinase-like proteins (such as YM-1) (22, 38, 39). Its key secretory profile is characterized by the production of substantial amounts of transforming growth factor-β (TGF-β), platelet-derived growth factor (PDGF), and insulin-like growth factor-1 (IGF-1) (40). Functionally, M2a is a core driver of tissue fibrosis: TGF-β is the most potent pro-fibrotic factor, directly activating fibroblasts to differentiate into myofibroblasts that secrete excessive collagen; PDGF is a potent mitogen for fibroblasts (41). Therefore, during the TED quiescent phase, M2a macrophages are considered the key drivers of orbital fibroblast activation, excessive collagen deposition, and ultimately pathological fibrosis, leading to irreversible damage such as restrictive strabismus and eyelid retraction (31).

M2b subtype: Known as the “regulatory type,” it can be induced by synergistic stimulation from immune complexes combined with LPS or IL-1 receptor agonists (22, 42, 43). Its unique cytokine secretion pattern involves high expression of IL-10 alongside the production of IL-1β and TNF-α (44). On the one hand, IL-10 suppresses excessive inflammation; on the other hand, it participates in the polarization of Th2 immune responses and the progression of certain chronic inflammatory models (45). This unique secretion profile confers a complex role in regulating immune responses, potentially participating in immune modulation during the active-quiescent transition phase of TED. It suppresses excessive inflammation while also contributing to the maintenance of chronic disease progression.

M2c subtype: Primarily induced by IL-10 or glucocorticoids, often referred to as “suppressive” or “repair” macrophages (46). They highly express CD163 and Mer tyrosine kinase (MerTK), with IL-10 and TGF-β as their signature secreted factors (47, 48). The core function of M2c lies in promoting inflammation resolution and tissue remodeling: They participate in extracellular matrix degradation and reconstruction through efficient efferocytosis of apoptotic cells and secretion of tissue inhibitors of metalloproteinases (TIMPs) and TGF-β. During TED repair, M2c contribute to the inflammatory resolution phase; however, persistent secretion of TGF-β could similarly shift toward promoting fibrotic effects (49, 50).

M2d subtype: Additionally, in certain specific contexts (such as the tumor microenvironment), M2d subtypes exist. These are induced by TLR antagonists, adenosine A2A receptor agonists, or IL-6, and participate in immunosuppression and angiogenesis by secreting IL-10 and vascular endothelial growth factor (VEGF) (51, 52). Its role in TED remains unclear, though it may be involved in abnormal angiogenesis during the quiescent phase or in the formation of an immunosuppressive microenvironment.

However, contemporary single-cell transcriptomics and network analysis reveal that these traditional classifications are not sharply delineated, independent entities, but rather characteristic nodes along a continuous, multidimensional functional spectrum (11). Transcriptome-based lineage models reveal that macrophage activation represents a continuous spectrum, with specific activation states determined by precise combinations and doses of multiple microenvironmental signals. This insight compels us to move beyond simplistic M1/M2 dichotomies and instead examine the dynamic role of macrophages in TED’s pathological progression through the lens of a functional continuum. In TED, the dynamic alterations in orbital microenvironmental signals serve as the fundamental driving force behind the systematic, continuous shift of macrophages along this functional continuum.

## 3 Mechanisms of macrophage recruitment, origin, and activation in the TED orbital microenvironment

### 3.1 Chemokine-mediated monocyte recruitment network

Following successful recruitment of monocytes to the orbital tissues of thyroid eye disease (TED), their fate is not determined by a single signal but is instead regulated by the synergistic interplay of multiple cytokines, cell-cell contacts, and disease-specific signals within the local microenvironment. This intricate regulatory network governs the clonal expansion, tissue colonization, and functional polarization of macrophages, ultimately shaping them into the core effector cells driving chronic inflammation and tissue remodeling in TED (53).

The CCL2/CCR2 axis is widely recognized as the most central signaling pathway in the process of monocyte recruitment (54). In the TED pathological environment, activation of this pathway exhibits a multi-cell-origin characteristic: *In vitro* study indicate that extraocular muscle cells from TED patients can secrete the CCL2 chemokine under the influence of PPARγ-regulated cytokines (55). Under the stimulation of inflammatory mediators such as histamine and IL-1β, as well as autoimmune signals, orbital fibroblasts upregulate CCL2 expression through pathways including NF-κB (55, 56). Single-cell RNA sequencing further reveals that both the RASD1+ LPF fibroblast subpopulation and ACKR1+ endothelial cells present in TED contribute to the high expression and enhanced signaling of CCL2, forming a multicellular secretory network (57). Circulating CCR2+ classical monocytes (CD14++ CD16−) exhibit high sensitivity to CCL2 chemotactic gradients and demonstrate upregulation of CCR2 expression in patients with autoimmune thyroid disease (58). These cells are specifically recruited to orbital tissues, where they differentiate into tissue-resident macrophages under the influence of local microenvironmental factors such as macrophage colony-stimulating factor (M-CSF), becoming early effector cells in orbital immune inflammation.

Beyond the CCL2/CCR2 axis, other chemokines also form a synergistic regulatory network. CXCL10, as a representative inducible by type I interferons (such asIFN-γ), is considered one of the key molecular bridges linking systemic autoimmunity with local orbital inflammation. In patients with Graves’ disease and TED, CXCL10 levels are significantly elevated in peripheral blood and extraocular muscle tissues, and are closely correlated with disease activity (55, 59, 60). CXCL10 is secreted in large quantities by orbital fibroblasts and other cells under IFN-γ induction. By binding to CXCR3, it recruits Th1 lymphocytes, forming an inflammatory positive feedback loop that exacerbates tissue damage (61, 62). The CXCL12/CXCR4 pathway participates in the directed migration of fibroblasts and the transport of T cells/macrophages, and interacts with TSHR signaling (61). Additionally, chemokines such as CCL5, CCL7, CXCL9, and CXCL11 collectively participate in the synergistic chemotaxis of monocytes and lymphocytes, while PPARγ activation can partially suppress the excessive activation of this inflammatory chemokine network (60, 63, 64).

### 3.2 Synergistic effects of clonal expansion and survival signals

The local proliferation of macrophages within orbital tissues is a critical mechanism for sustaining and amplifying inflammation. Macrophage colony-stimulating factor (CSF-1/M-CSF) plays a central role in this process, being persistently overexpressed by activated orbital fibroblasts in the orbital adipose tissue and fibrotic regions of TED patients (65). CSF-1 primarily drives cell proliferation by activating the MEK/ERK signaling pathway: activated ERK1/2 phosphorylates and activates downstream transcription factors (such as c-Myc and Elk-1), thereby upregulating cyclin D1 expression and propelling macrophages through the G1/S checkpoint to achieve local clonal expansion (66–68). The PI3K/Akt/mTOR pathway acts in concert with this mechanism, serving as a pivotal regulator of cell survival and metabolic reprogramming. On one hand, it promotes macrophage survival by suppressing apoptotic signals; on the other hand, it enhances protein and lipid synthesis through activation of mTORC1, thereby providing essential biosynthetic support for sustained cellular existence and functional execution (66, 69, 70).

High-throughput proteomics and immunohistochemical analysis of orbital connective tissue revealed that macrophages residing in orbital tissues establish stable connections with matrix cells through altered integrin expression profiles (such as upregulation of β3 integrin), while acquiring tissue-specific metabolic characteristics. This provides a structural foundation for their long-term survival and functional execution (71). Additionally, the combined action of factors such as macrophage chemotactic protein-1 and transforming growth factor-β contributes to the persistence of orbital inflammation (72).

### 3.3 Intersection of TED-specific activation signals with classical immune pathways

Beyond universal amplification signals, macrophage activation in TED undergoes multi-level signal integration regulation, with the TSHR/IGF-1R functional axis constituting a unique pathological pathway distinct from other autoimmune diseases (73–75). *In vitro* studies confirm that fibroblasts and macrophages in orbital tissue constitutively express TSHR and IGF-1R, with both receptors forming functional complexes on the cell membrane surface (76–80).

TSHR stimulating antibodies not only directly induce mild macrophage activation, but more importantly significantly lower the activation threshold of IGF-1R through allosteric regulation (81–83). Orbital macrophages constitutively express TSHR and IGF-1R. TRAb binds to TSHR and forms a functional complex with IGF-1R, synergistically activating downstream Ras/Raf/MEK and PI3K pathways. This mechanism directly explains why orbital inflammation can persist independently even after thyroid function normalizes. The aforementioned TED-specific mechanisms intersect with classical immune activation pathways, collectively forming a potent inflammatory amplification network.

## 4 Dynamic evolution of the macrophage functional state continuum in the pathological process of TED

Within the orbital microenvironment, the functional state of macrophages manifests as a dynamic functional continuum rather than a static M1/M2 phenotype. During active TED, pro-inflammatory signals drive macrophages toward the pro-inflammatory end of the functional spectrum, dominating the inflammatory response. As disease progresses, microenvironmental signals shift toward pro-repair/fibrotic pathways, driving the overall functional spectrum of macrophages toward the fibrosis-promoting end. This facilitates extracellular matrix deposition through the secretion of mediators such as TGF-β, thereby governing the fibrotic process. Thus, the evolution of disease stages fundamentally represents a systematic shift in the functional spectrum of macrophages under dynamic microenvironmental regulation (12, 13, 84) (**Table 1**).

**Table 1:** Pathogenesis of thyroid eye disease (TED): dynamic evolution of the macrophage functional state continuum.

Disease stage	Core event	Key cells/molecules	Molecular regulatory pathways and key factors
active phase	Initiation of the Inflammatory Cascade	M1-like macrophages (functional lineage converging toward the pro-inflammatory end), activated orbital fibroblasts, Th1 cells, DAMPs, IFN-γ, TNF-α, IL-1β, IL-6	TLR4/NF-κB pathway: DAMPs activation drives IL-6, TNF-α, and IL-1β release. JAK-STAT1 pathway: Activated by IFN-γ, upregulates iNOS and CD86.
	The Establishment of the Inflammatory Microenvironment and Tissue Injury	Promoting inflammatory function: Macrophages, activated orbital fibroblasts, TNF-α, IL-1β, MMP-9, MMP-2, NO	NF-κB/MAPK pathway: TNF-α/IL-1β activates the inflammatory response in fibroblasts. High expression of MMP-9/MMP-2 degrades the extracellular matrix (ECM); iNOS production of NO leads to oxidative stress and edema.
	Initiation and Maintenance of Adaptive Immunity	M1-like macrophages (as APCs), CD4+ T cells (Th1/Th17)	High expression of MHC-II, CD80/CD86, IL-12/IL-23 promotes Th1/Th17 differentiation, forming a positive feedback loop of IFN-γ/IL-17.
Dynamic transition from the active phase to the quiescent phase	Cytokine Profile Conversion	Macrophages (functional transition from pro-inflammatory to pro-repair/fibrotic), Th2 cells, regulatory T cells, IL-4, IL-13, IL-10, TGF-β, SPMs, α-MSH	The STAT6 pathway is activated, suppressing pro-inflammatory programs and initiating repair genes.
	Induction of Hypoxic Microenvironments	M2-like macrophages, hypoxia-inducible factor (HIF-2α)	HIF-2α promotes the expression of Arg-1, VEGF, and other factors, driving a functional state shift.
	Core Drivers of Metabolic Restructuring	Macrophages (metabolic state transition, PPARγ, PGC-1β)	Glycolysis to oxidative phosphorylation/fatty acid oxidation. PPARγ/PGC-1β pathway: Promotes fatty acid oxidation and mitochondrial biogenesis, coupling metabolic reprogramming with pro-fibrotic transcriptional programs.
Quiescent phase	Fibrogenic functional state drives tissue remodeling	Macrophages (functional lineage locked toward the pro-fibrotic pathway), orbital fibroblasts, myofibroblasts, TGF-β, PDGF	TGF-β/Smad & p38 MAPK pathways: TGF-β1 secreted by M2a cells activates fibroblasts, inducing their transdifferentiation into α-SMA+ myofibroblasts. PDGF pathway: Potently promotes fibroblast proliferation.
	Interactions of Specific Cell Subpopulations and the Maintenance of Fibrosis	PDGFRα^+^DPP4^+^ fibroblast subpopulation – M2-like macrophages	GAS6-AXL pathway: Macrophage-mediated enhancement of fibroblast fibrotic activity.
	Metabolic remodeling supports persistent fibrosis	M2-like macrophages, fibroblasts, PPARγ	Sustained activation of the PPARγ pathway: Drives fatty acid oxidation, providing energy and substrates for ECM synthesis, achieving metabolic-functional integration.

### 4.1 Active phase: inflammatory cascade dominated by pro-inflammatory macrophage state

During the TED phase, the innate immune response centered on M1-like macrophages triggers a complex inflammatory cascade. The disease initiates with tissue damage induced by autoimmune attacks, releasing endogenous danger signals (DAMPs) such as hyaluronan fragments (70, 85). These signals strongly activate the nuclear factor κB (NF-κB) signaling pathway by binding to Toll-like receptor 4 (TLR4) on the surface of macrophages, thereby driving the explosive release of key proinflammatory cytokines such as interleukin-6 (IL-6), tumor necrosis factor-α (TNF-α), and interleukin-1β (IL-1β) (12, 86, 87). Concurrently, interferon-γ (IFN-γ) derived from Th1 lymphocytes, via the JAK-STAT1 pathway, drives a robust shift in the transcriptional program of macrophages toward the extreme region of the M1-like activation axis by upregulating macrophage marker molecules such as iNOS and CD86. This combination of stimuli shapes a cellular state characterized by high output of proinflammatory mediators (12). The establishment and consolidation of this proinflammatory phenotype relies on profound transcriptional and epigenetic remodeling. Key transcription factors such as NF-κB and STAT1 are synergistically activated, jointly binding to enhancer regions of genes like IL6 and TNF to directly drive their efficient transcription. These transcription factors recruit chromatin remodeling complexes like SWI/SNF and histone acetyltransferase HDAC4 to open chromatin at proinflammatory gene sites. H3K4me3, together with acetylation (H3K27ac) and chromatin accessibility, jointly shapes the epigenetic landscape of macrophages and defines their threshold for responding to environmental signals (88–91). This metabolic-epigenetic coupling, such as acetyl-CoA derived from glycolysis supporting histone acetylation, further consolidates the state of macrophages at the pro-inflammatory end of the functional continuum.

Macrophages at the extreme end of this functional spectrum establish and maintain the local inflammatory microenvironment through multiple mechanisms. First, the TNF-α and IL-1β they secrete activate orbital fibroblasts, inducing high expression of chemokines such as CCL2 and CXCL10 via the NF-κB and MAPK signaling pathways. This, in turn, recruits additional monocytes, forming a self-reinforcing positive feedback loop of inflammation (87, 92, 93). Secondly, under hypoxic conditions, M1-like macrophages exhibit enhanced TNF-α secretion capacity. This synergizes with the hypoxic environment to induce HIF-1α expression in TED orbital fibroblasts, thereby promoting the production of chemokines such as CCL2, CCL5, and CCL20. This process further recruits macrophages and other immune cells while driving tissue remodeling processes including adipogenesis (94). Regarding structural damage, pro-inflammatory macrophages predominantly expressing M1 phenotype degrade components such as collagen and elastin in the extracellular matrix (ECM) through high expression of matrix metalloproteinases (such asMMP-9 and MMP-2). This process disrupts tissue architecture and promotes fibroblast migration and proliferation, with their activity closely correlated to the degree of orbital tissue remodeling in TED patients (95, 96). Concurrently, pro-inflammatory macrophages produce large amounts of nitric oxide (NO) via iNOS, causing vasodilation, tissue edema, and oxidative stress. This leads to damage to lipids, proteins, and DNA, thereby further amplifying inflammation and tissue destruction (97, 98). Additionally, M1-like proinflammatory macrophages, acting as professional antigen-presenting cells, present self-antigens (such as IGF-1R or TSHR) to CD4+ T cells through high expression of MHC class II molecules and co-stimulatory molecules CD80/CD86. They also provide the second signal essential for full T cell activation, thereby initiating and sustaining an adaptive immune response against orbital tissues (82, 99–101). Activated Th1/Th17 cells secrete cytokines such as IFN-γ and IL-17, which in turn reactivate fibroblasts and macrophages, forming another positive feedback loop that perpetuates autoimmune attacks over the long term (102, 103). This series of pathological processes exhibits persistent and intensified characteristics, driven by the aforementioned consolidated pro-inflammatory transcriptional and epigenetic programs.

### 4.2 Dynamic transition from active to quiescent state: microenvironmental signal remodeling drives functional reorientation of macrophages

The transition of TED from the active phase to the quiescent phase is fundamentally driven by dynamic shifts in the orbital microenvironment’s stimulus signaling profile. This drives a systematic, continuous shift in the overall transcriptional program of macrophages along a functional continuum, rather than a simple “M1 to M2 conversion. “ During this process, sustained exposure to signals may induce stable epigenetic modifications, thereby partially “fixing” the new functional state, reducing cellular plasticity, and providing molecular memory for disease progression.

Cytokine Profile Shift: As disease progresses, the adaptive immune response shifts from Th1 dominance to Th2 bias (104, 105). Th2-derived cytokines (such as IL-4 and IL-13) and IL-10 levels produced by regulatory T cells significantly increase in the microenvironment, forming new and complex stimulus combinations with residual inflammatory signals. This leads to a gradual shift in the transcriptional regulatory network driving macrophages. Initially dominated by STAT1/NF-κB, which drives the early pro-inflammatory response, the network progressively shifts toward greater involvement of transcription factors such as STAT6, PPARγ, and KLF4. This transition initiates the suppression of certain pro-inflammatory pathways and activates the expression of genes associated with tissue repair (106, 107). Simultaneously, endogenous inflammation resolution programs are activated, with pro-resolving mediators (SPMs) further regulating inflammation resolution and tissue repair by limiting neutrophil infiltration and stimulating local macrophage function (108). Within this intricate regulatory network, endogenous melanocortin systems (such as α-MSH) have also been demonstrated to modulate macrophage reactivity. By suppressing excessive inflammatory responses, they exert protective effects across various pathological processes including cutaneous inflammation, joint diseases, and ischemia-reperfusion injury (109). *In vitro* study indicate that α-MSH significantly inhibits inflammation and pro-melanocortin POMC production in orbital fibroblasts associated with thyroid-related eye disease (110). This sustained signal exposure induces stable epigenetic remodeling, thereby “solidifying” the new functional state. Under repair-promoting signals, enhancer regions of repair-related genes (TGFB1, COL1A1) accumulate active histone marks (H3K27ac), while repressive DNA methylation at their promoter regions may be removed. These changes collectively enhance transcriptional activity and gene accessibility (111).

Hypoxic microenvironment induction effect: Rapidly proliferating fibroblasts and highly metabolically active inflammatory cells within the orbital cavity consume large amounts of oxygen, leading to the formation of localized hypoxic zones (94). In this process, the stable hypoxia-inducible factors HIF-1α and HIF-2α play a crucial role (112). In TED vitro studies indicate that HIF-2α (rather than HIF-1α) specifically promotes the expression of M2-associated genes (such as Arg-1 and VEGF) in macrophages, directly driving their polarization toward an M2-like phenotype (113, 114). This hypoxia-driven polarization pattern tightly links metabolic stress with immune remodeling.

The core driving role of metabolic reprogramming: The metabolic state of macrophages progressively shifts from aerobic glycolysis during the active phase to a state dominated by oxidative phosphorylation and fatty acid oxidation (115). Peroxisome proliferator-activated receptor gamma (PPARγ) and its coactivator PGC-1β serve as the core regulatory factors in this metabolic transition (116, 117). Pro-inflammatory signals and autoantibodies activate aerobic glycolysis and the mevalonate pathway in orbital fibroblasts, thereby supplying metabolic substrates for hyaluronan synthesis and adipogenic differentiation. while simultaneously intervening in histone acetylation (H3K27ac) and methylation (H3K4me3) modifications via acetyl-CoA and mevalonic acid metabolites. This establishes a ‘lipogenic permissive’ epigenetic landscape within the promoter regions of adipogenic genes (PPARγ) (118, 119). Once established, this metabolically driven epigenetic imprint enables fibroblasts to maintain a low-threshold response phenotype even after initial stimulus withdrawal. Simultaneously, it drives infiltrating macrophages away from the classical M1/M2 dichotomy towards a pro-inflammatory/pro-fibrotic hybrid continuum. This explains why TED exhibits a protracted, recurrent clinical course. Targeting the mevalonate pathway (statins) or epigenetic modifiers (HDAC/EZH2 inhibitors) can erase pathological epigenetic memory by remodeling chromatin accessibility, offering a novel therapeutic paradigm for TED that transcends conventional anti-inflammatory strategies (117, 120). Research on immune training demonstrates that following cessation of pro-inflammatory stimuli, metabolic nodes such as the mevalonate pathway continue to mediate immune memory via a persistent state of chromatin accessibility. Conversely, endotoxin tolerance characterizes an imprinting state exhibiting opposing functional properties (91). Furthermore, distinct macrophage subtypes exhibit differentiated iron metabolism patterns; conversely, iron metabolism can also regulate macrophage polarization and inflammatory output by influencing oxidative stress and cellular metabolic states (50, 121, 122).

### 4.3 Quiescent phase: macrophage states dominated by pro-fibrotic functions lock the fibrosis process

During the TED quiescent phase, the microenvironment is dominated by high levels of TGF-β, PDGF, and specific extracellular matrix components. This combination of stimuli locks the activation state of macrophages onto the M2-like activation axis, a pathway closely associated with tissue remodeling and fibrosis. Sustained signaling drives a shift in core transcriptional regulation: transcription factors including STAT6, SMAD3, and PPARγ are synergistically activated, collectively upregulating expression modules of pro-fibrotic genes (such asTGFB1, COL1A1, ARG1). At this stage, the functional continuum of macrophages distinctly tilts toward the pro-fibrotic lineage. Clinical evidence unequivocally demonstrates that the orbital tissues of stable GO patients are enriched with CD163+ M2-like macrophages, whose density significantly exceeds that of controls. Both *in vivo* and *in vitro* experiments confirm these cells as the primary source of transforming growth factor-β (TGF-β) (12). These macrophages in a pro-fibrotic state serve as the core effector cells driving irreversible fibrosis, exhibiting functional output characteristics highly similar to the M2a subtype in traditional classification. Their key pathogenic mechanism lies in serving as the primary cellular source of TGF-β1: by activating both the canonical Smad signaling pathway and the non-canonical p38 MAPK pathway in orbital fibroblasts, TGF-β1 effectively induces their transdifferentiation into α-smooth muscle actin (α-SMA)-positive myofibroblasts. This process constitutes the core cellular event of pathological fibrosis (85, 123, 124).

Notably, the fibrotic drive of M2-like macrophages is significantly enhanced by the synergistic secretion of platelet-derived growth factor (PDGF). As a potent mitogen, PDGF specifically promotes fibroblast proliferation to expand the effector cell population. Studies indicate that PDGF induces hyaluronan synthase 2 (HAS2) gene expression and hyaluronic acid (HA) production in orbital fibroblasts from GO patients. Furthermore, HA released by fibroblasts may further activate macrophages by directly stimulating their surface CD44 receptors or synergizing with chemokines, thereby forming a positive feedback loop that sustains and exacerbates fibrosis (125). Meanwhile, TGF-β1 directs these cells toward a highly secretory phenotype. PDGF-mediated cell proliferation synergistically amplifies TGF-β1-driven functional differentiation, ultimately leading to abnormal ECM accumulation and progressive tissue structural remodeling (50, 126). During the quiescent phase of the Tonic-Dystonic Eye Movement (TED), both macrophages and fibroblasts within the orbital cavity may be regulated by the PPARγ signaling pathway (127, 128). Activation of PPARγ not only promotes M2-like macrophage polarization, but also drives fatty acid oxidation and enhanced mitochondrial function. This process provides energy and biosynthetic precursors for sustained ECM synthesis and tissue remodeling, thereby tightly linking metabolic reprogramming with pro-fibrotic functions (129).

## 5 Dynamic network interactions between the macrophage functional state continuum and the orbital microenvironment

The functional regulation of macrophages is not an isolated process but rather deeply integrated as a core node within the dynamic operation of the orbital immune micro-network. This interaction follows a dynamic logic of “signal input-functional output-environmental remodeling”: the combination of signals within the local microenvironment shapes the functional spectrum of macrophages. In turn, through their secretome and cell-cell contacts, these macrophages continuously feedback and remodel the cellular composition and signaling landscape of the microenvironment, thereby forming a self-sustaining loop that drives the phased progression of disease.

### 5.1 Macrophages and orbital fibroblasts: core positive feedback loop driving fibrosis progression

The interaction between macrophages and orbital fibroblasts constitutes the core driving force propelling TED from inflammation to fibrosis. This process transcends linear intercellular signaling, fundamentally involving mutual induction, synergy, and amplification of functional states between the two cell types, thereby forming a self-sustaining pathological feedback loop.

During disease activity, macrophages with transcriptomes skewed toward pro-inflammatory (M1-like) extremes dominate. By secreting core inflammatory mediators such as IL-6 and TNF-α, they activate signaling pathways like NF-κB within fibroblasts. This not only directly causes tissue damage but, more critically, induces fibroblasts to highly express chemokines like CCL2. This creates a self-reinforcing loop that recruits more monocytes/macrophages, continuously amplifying the local inflammatory storm. At this stage, macrophages can directly regulate the inflammatory response of fibroblasts through the IL-6/sIL-6R signaling pathway (12).

As the disease progresses into the transition and quiescent phases, alterations in the microenvironmental signaling spectrum drive a comprehensive shift in the functional spectrum of macrophages toward a pro-fibrotic domain. At this stage, macrophages in a pro-fibrotic state (functionally analogous to the traditional M2a subtype) form a “fatal alliance” with fibroblasts, becoming the direct executors of fibrosis. The core mechanism of this alliance lies in the coordinated secretion of key fibrotic mediators: TGF-β powerfully drives fibroblast differentiation into myofibroblasts that secrete extracellular matrix through pathways such as Smad2/3 activation. Single-cell RNA sequencing studies reveal that CD163+ tissue-infiltrating macrophages regulate ferroptosis in TED orbital fibroblasts via the TGF-β/Smad2/3 signaling pathway, thereby promoting fibrosis and lipogenesis in orbital tissues (50). Macrophages are key sources of TGF-β and PDGF (including PDGF-AB and PDGF-BB subtypes). Mast cells, monocytes, and macrophages infiltrating orbital tissues can produce platelet-derived growth factor subtypes such as PDGF-AB and PDGF-BB, synergistically regulating the activation process of orbital fibroblasts (126). Notably, M2-like macrophages were identified as a core driver of fibrosis in the orbital microenvironment through interactions with PDGFRα^+^DPP4^+^ fibroblasts via the GAS6–AXL signaling pathway. Single-cell RNA sequencing data revealed that M2 macrophages further promote fibrotic processes in PDGFRα^+^DPP4^+^ fibroblasts through the GAS6–AXL pathway (13). Furthermore, the identification and validation of key genes associated with iron death in thyroid-associated ophthalmopathy revealed that M2-like macrophages may participate in regulating ACO1 expression in orbital fat-derived OFs (122). In the TED mouse model, early-infiltrating macrophages interact with antigen-specific pro-inflammatory T cells, inducing pathogenic anti-TSHR antibody production. This subsequently activates orbital fibroblasts/pre-adipocytes, triggering inflammation and extracellular matrix deposition (76). Simultaneously, multiple signaling pathways participate in integrative effects: beyond the aforementioned core pathways, under hypoxic conditions, co-culture of M1-like macrophages with orbital fibroblasts enhances adipogenic differentiation and adiponectin secretion, indicating that M1-like macrophage/fibroblast interactions drive persistent inflammation and tissue remodeling in TED (94). Macrophages can also induce orbital fibroblasts to adopt a pro-fibrotic phenotype by promoting hyaluronic acid synthesis and enhancing cellular contractility (125).

### 5.2 Macrophages and adipocytes: cross-regulatory network between metabolism and immunity

The bidirectional interaction between macrophages and orbital adipocytes serves as a pivotal hub regulating orbital tissue volume and inflammatory status. The core characteristic of this interactive network lies in its dynamic and environment-dependent nature, where the functional continuum of macrophages plays a decisive role. On one hand, the functional state of macrophages directly regulates the process of lipogenesis (125, 130). Macrophages in a pro-inflammatory functional state (with a transcriptional profile biased toward the M1-like axis) typically suppress the function of key adipogenesis transcription factors PPARγ and C/EBPα by secreting factors such as TNF-α and IL-1β, thereby inhibiting the differentiation and maturation of preadipocytes (131). Notably, under specific conditions such as hypoxic co-culture environments, M1-like macrophages can also enhance adipogenic differentiation and adiponectin secretion, suggesting that their effects are context-dependent (94). During the resolution or repair phase of inflammation, M2-like macrophages produce factors such as IL-10 and TGF-β. At low concentrations, TGF-β acts as a potent mitogen, strongly promoting the proliferation of orbital fibroblasts and adipocytes. It also upregulates IGF-1R on orbital fibroblasts, forming a positive feedback loop with TSHR signaling that amplifies fibroblast responsiveness to adipogenic signals (132). On the other hand, adipocytes are not passive targets but actively shape the functional lineage of macrophages by secreting adipokines. Leptin drives macrophages toward a pro-inflammatory state, enhancing their capacity to produce IL-6 and TNF-α, thereby forming a pro-inflammatory positive feedback loop; conversely, adiponectin promotes the shift of macrophages toward an anti-inflammatory/reparative state (133). In the orbital adipose tissue of TED patients, the imbalance between leptin and adiponectin levels may provide a key molecular basis for local inflammation and abnormal tissue remodeling by persistently affecting the functional localization of macrophages. This demonstrates that macrophages and adipocytes form a complex, bidirectional regulatory network through multiple signaling molecules, jointly participating in the pathological progression of TED (134–136).

### 5.3 Macrophages and T cells: a bidirectional loop guiding and regulated by adaptive immunity

The interaction between macrophages and T cells forms a critical bridge connecting innate and adaptive immunity, with its dynamic equilibrium directly determining the inflammatory characteristics and disease outcomes of TED. As antigen-presenting cells and cytokine sources, phagocytes play a guiding role in T cell differentiation. Pro-inflammatory macrophages drive the differentiation of naive CD4^+^ T cells toward Th1 and Th17 subsets by presenting tissue-specific antigens (such asTSHR) and secreting IL-12 and IL-23 (76). Activated Th1 cells secrete IFN-γ, while Th17 cells secrete IL-17. These factors not only directly exacerbate tissue damage and fibrosis but also serve as one of the most potent signals amplifying the proinflammatory state of macrophages. This establishes a positive feedback loop between “Th1/Th17 cells and proinflammatory macrophages,” driving the immunopathology during disease activity (72, 102, 137). Conversely, as the disease progresses, Th2 cells drive the functional spectrum of macrophages toward a pro-repair/fibrotic state by secreting IL-4 and IL-13. These pro-fibrotic macrophages subsequently activate fibroblasts and promote collagen deposition by secreting factors such as TGF-β and PDGF, thereby establishing a fibrosis-driving axis of “Th2-pro-fibrotic macrophages-fibroblasts” during the chronic phase. Thus, the disease process of TED fundamentally represents a dynamic tug-of-war and cyclical shift in dominance between the “Th1/pro-inflammatory macrophage axis” and the “Th2/pro-fibrotic macrophage axis (37).

### 5.4 Macrophages and B cells: the chronic engine sustaining innate humoral immunity

The interaction between macrophages and B cells is a crucial mechanism that directs innate immune responses toward persistent humoral immunity and explains the chronic characteristics of TED. Activated macrophages, particularly those in a pro-inflammatory state, provide essential co-stimulatory signals for B cell survival, proliferation, and terminal differentiation within the local orbital microenvironment or associated lymphoid tissues. This occurs through the production of key cytokines such as B cell activating factor (BAFF) and interleukin-6 (IL-6). BAFF is a critical survival factor for B cells, and its abnormally high expression is closely associated with autoimmunity. IL-6, meanwhile, is the core cytokine driving the final differentiation of B cells into antibody-secreting plasma cells (74). Based on this differentiation pathway, long-lived plasma cells are formed and continuously produce autoantibodies against TSHR, becoming the core executors of TED pathogenesis (138). Once formed, these plasma cells can secrete antibodies persistently, even without ongoing antigenic stimulation, thereby directly explaining the chronic and recurrent clinical characteristics of TED (139, 140). Additionally, plasma cells and B cells can further sustain the activated state of T cells and macrophages through antigen presentation and cytokine secretion, ultimately forming a self-perpetuating, difficult-to-break “macrophage-T cell-B cell” triangular inflammatory network that drives the continuous progression of the disease.

## 6 Therapeutic implications and future outlook

Based on the core regulatory role of the macrophage functional state continuum in the pathological progression of thyroid eye disease (TED), intervention strategies targeting its plasticity and the accompanying immunometabolic-transcriptomic-epigenetic regulatory networks demonstrate the potential for precision therapy beyond traditional immunosuppression. While conventional therapies can nonspecifically suppress inflammation, they struggle to reverse fibrosis progression and fail to precisely modulate the dynamic functional states of macrophages across different disease stages. This chapter systematically examines the limitations of current treatments. Guided by the principle of “reprogramming macrophage functional lineages rather than simple elimination,” it analyzes potential therapeutic strategies targeting macrophage functional state transitions. Finally, it proposes key directions for future research (**Table 2**).

**Table 2:** Macrophage-based therapeutic approaches for thyroid eye disease (TED): limitations and potential side effects.

Treatment strategy categories	Specific targets/drugs	Macrophage-related mechanisms of action	Limitations	Potential side effects
I. Current Conventional Treatments	Glucocorticoids	Non-specifically inhibits the activation of immune cells such as macrophages and the release of inflammatory cytokines (TNF-α, IL-1β, IL-6), primarily suppressing acute inflammation driven by M1-type macrophages.	It is largely ineffective against the chronic fibrotic microenvironment maintained by M2-like macrophages; long-term use is prone to tolerance.	Iatrogenic cataracts, mood disorders, glaucoma
	Immunosuppressants (such as cyclosporine, methotrexate, mycophenolate mofetil)	By suppressing T and B lymphocyte activation and proliferation, it indirectly reduces macrophage activation signals (such as IFN-γ) and recruitment.	Slow onset of action, with limited ability to reverse established fibrotic pathological structures; significant interindividual variability in therapeutic response.	Bone marrow suppression; impaired liver and kidney function; gastrointestinal reactions; cyclosporine may cause gingival hyperplasia and hirsutism; risk of malignancy.
	B-cell targeted drugs (such as rituximab)	Depletion of CD20^+^ B cells reduces autoantibody production and antigen presentation, thereby indirectly weakening T cell-dependent macrophage activation.	Cannot eliminate differentiated long-lived plasma cells; has a delayed and incomplete effect on existing TRAb levels; ineffective against non-B cell-dependent inflammatory pathways.	Infusion reactions, infection, persistent hypogammaglobulinemia.
II. Breakthrough Targeted Therapies.	IGF-1R inhibitors (such as Teprotumumab).	Direct effects: Blocking endogenous IGF-1R signaling in macrophages to interfere with their function and polarization. Indirect effects: Inhibiting T cell activation and the production of proinflammatory factors (such asIFN-γ, TNF-α) to reduce signals driving M1 polarization; suppressing fibroblast production of inflammatory factors (such asIL-6, IL-8) to improve the macrophage microenvironment.	For late-stage quiescent lesions primarily characterized by muscle contracture and adipose fibrosis, improvement is limited; some patients exhibit primary or secondary non-response.	Muscle cramps, nausea, diarrhea, hair loss; reversible hearing loss; hyperglycemia; rare severe hypersensitivity reactions.
	IL-6 Receptor Inhibitor (Tocilizumab).	Competitively binds soluble and membrane-bound IL-6 receptors, blocking IL-6-mediated activation and proliferation of macrophages, T cells, and B cells, thereby suppressing acute-phase reactions.	Primarily indicated for the active phase of inflammation; there is insufficient evidence of direct effects on fibrosis itself. Long-term efficacy and optimal treatment duration remain to be determined.	Risk of infection and liver injury; Neutropenia; Hyperlipidemia; Risk of gastrointestinal perforation when used concomitantly with NSAIDs.
III. Other Potential Strategies Targeting Macrophages				
1. Targeted Recruitment and Amplification.	CCL2/CCR2 axis antagonists (such as PF-04136309).	Blocking the CCR2 receptor on monocyte surfaces inhibits their chemotaxis toward CCL2-high-expressing diseased periorbital tissues, thereby reducing infiltrating macrophage precursors at the source.	It is ineffective against resident macrophages already settled within the tissue or cells recruited via other chemotactic pathways; it may interfere with physiological tissue repair and anti-tumor immune surveillance.	Abnormal liver function, risk of infection.
	CSF-1/CSF-1R inhibitors (such as Pexidartinib).	Blocking colony-stimulating factor 1 receptors inhibits macrophage survival, proliferation, and differentiation, directly depleting macrophages within tissues.	The lack of cell subset selectivity results in the non-specific clearance of macrophage subpopulations critical for maintaining tissue homeostasis and physiological repair processes, posing risks of immune microenvironment imbalance and excessive intervention.	Skin hyperpigmentation, rash, hair discoloration; peripheral edema; fatigue, nausea; hepatotoxicity; hematologic abnormalities.
2. Regulation of Polarization and Function.	PPARγ agonists (such as pioglitazone).	Inhibits TNF-α-induced upregulation of TGF-β, HAS, and HA, promotes macrophage polarization toward an anti-inflammatory, pro-repair M2-like phenotype, and suppresses pro-inflammatory pathways such as NF-κB.	In complex fibrotic microenvironments, pro-fibrotic M2 subpopulations (such as TGF-β-secreting macrophages) may be inadvertently amplified.	Congestive heart failure, weight gain, risk of fractures, risk of bladder cancer. proadipogenic functions in the orbit might worsen the disease
	TGF-β pathway inhibitors (such as Galunisertib).	Selectively inhibiting TGF-β receptor I kinase blocks its downstream Smad signaling pathway, disrupting the core pro-fibrotic dialogue between M2-like macrophages and fibroblasts.	TGF-β plays a crucial role in maintaining immune tolerance, suppressing tumorigenesis, and promoting wound healing; long-term systemic inhibition carries significant risks.	Cardiac toxicity; bleeding tendency; fatigue, nausea; potential teratogenicity.
	JAK/STAT inhibitors (Tofacitinib, Baricitinib).	Broad-spectrum inhibition of JAK kinases blocks signaling pathways for multiple cytokines, including IL-4, IL-6, IL-13, and IFN-γ, thereby comprehensively regulating macrophage polarization and function.	Pathway inhibition is broad-spectrum and lacks precision targeting of TED-specific pathological pathways; it may affect multiple hematopoietic processes and immune functions.	Risk of infection, thrombosis, and malignant tumors; hyperlipidemia; gastrointestinal perforation.
	AXL/GAS6 Axial Suppressor (Bemcentinib).	Blocking the binding of the tyrosine kinase receptor AXL to its ligand GAS6 specifically disrupts the positive feedback pro-fibrotic circuit between M2-like macrophages and pathological fibroblasts.	Current research primarily focuses on oncology and pulmonary fibrosis, with limited clinical data available in TED; AXL plays physiological roles in platelet aggregation and innate immunity.	Risk of bleeding; fatigue, loss of appetite; liver damage.
	PD-1/PD-L1 inhibitors.	Theoretically, releasing immune checkpoint inhibitors on T cells and macrophages may restore the function of exhausted immune cells, but the specific effects on the TED fibrosis microenvironment remain highly complex and largely unknown.	Its use in autoimmune diseases carries significant risks, potentially triggering or exacerbating autoimmune reactions, including subacute thyroiditis or uveitis.	Multiple organ immune-related adverse reactions, which can be fatal in severe cases.
3. Targeting Intercellular Communication.	AMPK agonist (metformin).	Activate AMP-activated protein kinase to promote oxidative phosphorylation metabolism in macrophages, driving their transition to an anti-inflammatory phenotype while inhibiting NLRP3 inflammasome activation.	As a repurposed drug, its specific efficacy in TED remains to be confirmed by high-quality clinical studies; its effects are indirect and multifaceted.	Gastrointestinal reactions, lactic acidosis; impaired vitamin B12 absorption.
	PI3Kγ Inhibitor (Duvelisib).	Inhibiting phosphoinositide 3-kinase gamma (PI3Kγ) reverses tumor/fibrosis-associated macrophages from an immunosuppressive/pro-fibrotic M2-like phenotype to a pro-inflammatory/anti-fibrotic M1-like phenotype.	May exacerbate localized inflammatory responses in the short term; its effects on different macrophage subpopulations are complex, making therapeutic outcomes difficult to predict.	Severe diarrhea, colitis; liver toxicity; pneumonia and other infections; rash, allergic reactions.
4. Interventions in Metabolism and Mortality.	Ferrostatin-1 (Liproxstatin-1).	Inhibits iron-dependent lipid peroxidation through antioxidant mechanisms, thereby blocking fibroblast activation and fibrosis progression triggered by macrophage ferroptosis.	Currently confined to the basic research stage, drug stability, targeted *in vivo* delivery, and long-term safety pose significant challenges.	It is currently unclear.
5. Targeting extracellular matrix remodeling.	Matrix Metalloproteinase (MMP) Inducers/Activators.	By modulating the phenotype of cells such as macrophages, or through direct administration of drugs, enhance their ability to degrade excessively deposited extracellular matrix components like collagen.	The activity of MMPs must be precisely regulated; excessive activation may lead to disruption of normal tissue architecture and degradation of the vascular basement membrane, triggering hemorrhage.	Pain, bleeding, swelling; affecting wound healing and embryonic development.

### 6.1 Limitations of current conventional therapies and interpretation from a macrophage perspective

Current standard treatments for thyroid-associated eye disease (TED) primarily rely on glucocorticoids and immunosuppressants. While these drugs effectively suppress acute inflammatory responses dominated by M1-like macrophages, they have limited efficacy against established M2-like macrophage networks and the fibrotic microenvironment they maintain. This mechanism explains why existing therapies demonstrate significant efficacy during active inflammatory phases but yield suboptimal results for fibrotic lesions in quiescent states. From the perspective of macrophage plasticity, conventional strategies primarily intervene in M1 polarization during the inflammatory phase while failing to effectively regulate M2 polarization during the repair/fibrosis phase, thereby hindering the reversal of fibrosis progression.

Additionally, B-cell-targeted drugs such as rituximab can reduce the generation of new plasma cells but are ineffective against long-lived plasma cells. This phenomenon is closely associated with the self-perpetuating inflammatory network involving macrophages, T cells, and B cells observed in TED (141). The persistent activation of this network further highlights the limitations of single-target therapies, underscoring the need for future integrated therapeutic strategies targeting multiple cell types and signaling pathways.

### 6.2 Regulation of macrophage function by breakthrough targeted therapies

Targeting insulin-like growth factor-1 receptor (IGF-1R) represents a key therapeutic strategy for thyroid-associated eye disease (TED). Animal study indicate that the IGF-1R inhibitor linsitinib prevents autoimmune hyperthyroidism in experimental Graves’ disease (GD) models by mitigating pathological thyroid changes and suppressing T-cell infiltration (demonstrated by CD3 staining) during early stages. In the late stages of the disease, it primarily acts on orbital tissues, significantly reducing immune infiltration of T cells (CD3+) and macrophages (F4/80+, TNF-α+) within the orbit (142).

Teprotumumab, as a potent IGF-1R antagonist, has achieved clinical success, representing a breakthrough advancement in the treatment of TED (143, 144). Its mechanism of action is closely linked to the regulation of macrophage function, manifesting at both direct and indirect levels. Firstly, IGF-1R is overexpressed in orbital fibroblasts from TED patients and in various immune cells, including macrophages. *In vitro* study confirm that activated M2-like macrophages not only secrete IGF-1 but also express IGF-1R, thereby establishing an autocrine and paracrine feedback loop (145). Therefore, Teprotumumab by blocking IGF-1R signaling, not only directly inhibits fibroblast activation but may also directly interfere with the functional state and polarization of macrophages themselves (146). Secondly, this drug may also regulate macrophages via indirect pathways. It effectively suppresses T lymphocyte activation and the production of pro-inflammatory cytokines such as IFN-γ and TNF-α, which serve as key inducing signals driving macrophage polarization towards the M1 phenotype (147). Moreover, Teprotumumab also diminishes the capacity of orbital fibroblasts to produce inflammatory mediators such as IL-6 and IL-8 under TSH stimulation, thereby improving the microenvironment surrounding macrophages and indirectly promoting their transition from a pro-inflammatory phenotype to an anti-inflammatory/repair phenotype (148).

Tocilizumab, as a humanized monoclonal antibody targeting the IL-6 receptor, binds with high affinity to both soluble and membrane-bound forms of the IL-6 receptor, thereby systemically blocking IL-6’s canonical cis and trans signaling pathways (149). This precise intervention exerts multi-level regulation on macrophage function: firstly, it directly disrupts the IL-6/JAK/STAT3 signaling axis—a key driver of M1-like macrophage polarization—inhibiting their pro-inflammatory phenotype and potentially promoting a shift towards an anti-inflammatory/reparative phenotype (M2-like); Secondly, it markedly reduces the macrophage’s intrinsic capacity to produce IL-6 and other inflammatory mediators, disrupting the local positive feedback loop of the ‘cytokine storm’; Furthermore, by indirectly inhibiting IL-6-dependent Th17 cell differentiation and autoantibody production, it reduces upstream activation stimuli for macrophages (143). Clinical studies demonstrate that tocilizumab rapidly improves clinical activity scores, exophthalmos, and quality of life, proving particularly effective in patients resistant to glucocorticoids (150, 151). This therapeutically confirms the central role of the IL-6-macrophage axis in the pathogenesis of TED. Consequently, tocilizumab represents a profound shift in therapeutic strategy from non-specific immunosuppression towards targeted modulation of macrophage function, thereby blocking the disease progression pathway by remodeling the ocular immune microenvironment.

### 6.3 Other potential therapeutic strategies targeting macrophages

Beyond targeted therapies for IGF-1R and IL-6R, a series of innovative therapeutic strategies are being actively explored to intervene at distinct stages of macrophage recruitment, polarization, function, and metabolism. This approach stems from an in-depth understanding of the complex role macrophages play in the pathogenesis of TED. These strategies aim to intervene more precisely at different disease stages, drawing upon significant advances achieved in research on other fibrotic and inflammatory diseases.

#### 6.3.1 Recruitment and expansion of targeted monocytes/macrophages

In the early stages of disease, inhibiting the recruitment of monocytes to orbital tissues represents an upstream strategy for blocking the inflammation-fibrosis cascade. The CCL2/CCR2 axis constitutes the core pathway mediating monocyte chemotaxis; blocking this axis effectively reduces macrophage infiltration within lesions. Furthermore, the CSF-1/CSF-1R signaling pathway, as a key regulator of macrophage survival, proliferation, and differentiation, represents another critical intervention target. Inhibiting CSF-1R directly limits the expansion and maintenance of macrophages within tissues, thereby controlling their pathological effects at the cellular source (152). In studies of hepatic fibrosis, the small-molecule CCR2 antagonist PF-04136309 significantly reduced the number of activated hepatic stellate cells and macrophages in preclinical models, thereby delaying the progression of fibrosis (153). Similarly, in atherosclerosis research, CCR2 inhibitors have demonstrated potential for reducing inflammation within plaques (154, 155). Inhibitors targeting CSF-1R have also demonstrated efficacy in reducing pathological macrophage accumulation and improving disease progression across multiple fibrotic and inflammatory models. These findings provide robust theoretical support for TED treatment strategies. However, given the physiological roles of the CCL2/CCR2 axis and the CSF-1/CSF-1R pathway in tissue repair and immune surveillance, such interventions require precise timing to avoid adversely affecting physiological repair processes.

#### 6.3.2 Regulation of macrophage phenotypic polarization and function

In chronic immune-mediated diseases such as thyroid eye disease (TED), macrophages do not reside in static M1 or M2 polarized states but exist within a dynamic, continuous functional continuum. Their phenotype, metabolism, and function undergo continuous adjustment and remodeling in response to signals from the local microenvironment. Precise regulation of this continuum has emerged as a frontier strategy for intervening in disease progression. Specifically:

PPARγ agonists (such as pioglitazone) activate the nuclear transcription factor PPARγ, thereby not only directly driving the phenotypic shift of macrophages towards an anti-inflammatory, pro-repair ‘alternative activation’ state, but also suppressing classical pro-inflammatory signaling pathways such as NF-κB. This systematically remodels the macrophage gene expression profile at the transcriptional level, enhancing their phagocytic clearance of apoptotic cells and promoting the resolution of inflammation (156, 157). TGF-β pathway inhibitors (such as Galunisertib) act at the opposite end of the continuum, preventing macrophages from excessively shifting towards a pro-fibrotic phenotype during the late stages of chronic inflammation. Whilst TGF-β is a crucial reparative signal, its sustained activation drives macrophages to secrete substantial pro-fibrotic mediators (such as PDGF and CTGF) and activates orbital fibroblasts. Inhibiting this pathway prevents the functional shift of macrophages from repair to pathological fibrosis. For instance, the TGF-β receptor I inhibitor Galunisertib has demonstrated synergistic anti-fibrotic and anti-tumor effects in clinical trials for pancreatic cancer and glioblastoma, with confirmed capacity to reverse tissue fibrosis (158–160). Similarly, PDGF receptor inhibitors such as imatinib effectively suppress fibroblast proliferation and reverse fibrotic progression in systemic sclerosis and pulmonary fibrosis models, providing evidence for cross-disease therapeutic strategies in TED (161, 162).

JAK/STAT inhibitors (such as tofacitinib and baricitinib) provide broad-spectrum cytokine signaling interception capabilities. Multiple key cytokines driving macrophage differentiation towards distinct functional states (such as IFN-γ, IL-6, GM-CSF, etc.) transmit signals via the JAK-STAT pathway (163). Therefore, inhibiting this pathway can simultaneously block multiple polarization signals, thereby neutralizing the functional state of macrophages along a continuum and comprehensively suppressing their pathological activation.

In summary, these drugs meticulously intervene in the dynamic equilibrium of macrophages along the functional continuum through distinct mechanisms, including nuclear receptor transcriptional regulation, inhibition of key signaling pathways, and metabolic reprogramming. This provides a multidimensional, synergistic pharmacological strategy for developing TED-targeted therapies based on macrophage functional remodeling.

#### 6.3.3 Targeting intercellular communication

In the pathological progression of thyroid eye disease (TED), targeting the communication networks between immune cells has emerged as a novel strategy for modulating the functional state continuum of macrophages. Within the chronic inflammatory environment of TED, sustained activation of the AXL/GAS6 signaling pathway may drive macrophages towards an intermediate state polarization that exhibits both pro-fibrotic and immunosuppressive properties, thereby promoting the transition from tissue repair to pathological fibrosis (164). The interaction between GAS6 and AXL forms a positive feedback loop that continuously amplifies fibrotic signaling pathways (165). Bemcentinib inhibits this axis, thereby preventing the shift of macrophages towards this pathological functional phenotype and restoring their normal responsiveness to inflammatory environments. Inhibitors targeting this pathway, such as BGB324, have demonstrated safety and efficacy in preclinical studies and tumor clinical trials. They specifically block interactions between M2 macrophages and PDGFRα+DPP4+ fibroblasts, representing a novel therapeutic strategy precisely targeting the fibrotic microenvironment (13, 166). PD-1/PD-L1 inhibitors, however, target another critical immune checkpoint. This pathway may transmit inhibitory signals in TED through the interaction between PD-L1 on antigen-presenting cells such as macrophages and PD-1 on T cells. This not only suppresses T cell function but may also indirectly induce macrophages into a dysfunctional immunosuppressive state, thereby hindering the effective clearance of inflammation (167). Therefore, blocking PD-1/PD-L1 can release this inhibition and reshape the immune microenvironment, potentially prompting macrophages to rebalance from a state of excessive suppression or dysfunction towards a more effector-functional anti-inflammatory or homeostatic phenotype.

#### 6.3.4 Interfering with macrophage metabolism and novel cell death pathways

The dynamic positioning of macrophages along a functional state continuum is tightly coupled with profound reprogramming of their metabolic pathways: activation states biased towards the pro-inflammatory end of the continuum (characterized by high levels of pro-inflammatory cytokine production) primarily rely on glycolysis as their rapid energy supply foundation, whereas states shifting towards the anti-inflammatory and reparative end of the continuum increasingly depend on efficient oxidative phosphorylation to meet their long-term, steady-state functional demands (168). Based on this metabolic-functional coupling, regulating macrophage metabolic reprogramming has emerged as a novel strategy for modulating their polarization and function. Taking the AMPK agonist metformin as an example, it promotes the M2-like phenotype conversion of macrophages in obesity and diabetes models by enhancing oxidative phosphorylation and inhibiting inflammasome activation, thereby improving insulin sensitivity (169). Moreover, the macrophage replacement activation induced by long-term metformin intervention also demonstrated neuroprotective effects in an experimental stroke model (170). These findings suggest that metformin may offer dual therapeutic value for patients with thyroid eye disease who also have metabolic disorders such as diabetes. Beyond AMPK, other key metabolic nodes have been extensively studied. For instance, PI3Kγ is a pivotal signaling molecule regulating M2 polarization. Duvelisib (IPI-145) is an orally administered dual inhibitor of the PI3K-δ/γ subunits. vitro studies indicate that in mouse models transplanted with tumors derived from patients with peripheral T-cell lymphoma (PTCL), duvelisib treatment reverses tumor-associated macrophages from an immunosuppressive M2-like phenotype to a pro-inflammatory M1-like phenotype (171). This further demonstrates that targeting macrophage metabolism is an effective means of remodeling their functional phenotype and intervening in disease progression.

Moreover, ferroptosis, an iron-dependent form of regulated cell death, has in recent years been recognized as a key mechanism by which M2-like macrophages modulate fibroblast function.

Studies in renal and pulmonary fibrosis have revealed that M2-like macrophages can enhance fibroblast susceptibility to ferroptosis by secreting specific inducers (such as GPX4 inhibitors) or depleting intracellular protective substances (such as glutathione). This unexpectedly promotes fibroblast activation and proliferation, thereby accelerating the fibrotic process (172). Based on this mechanism, ferroptosis inhibitors such as Ferrostatin-1 and Liproxstatin-1 have demonstrated significant anti-fibrotic effects in multiple animal models of fibrosis. Their action works by protecting fibroblasts from ferroptosis, thereby halting the progression of the disease (173, 174). Concurrently, our team’s latest research has further demonstrated in Thyroid eye disease (TED) that CD163+ tissue-infiltrating macrophages directly regulate the ferroptosis process in orbital fibroblasts by activating the TGF-β/Smad2/3 signaling pathway (50). These findings collectively demonstrate that targeting the ferroptosis pathway on the macrophage-fibroblast axis reveals a highly promising new intervention target for the refractory fibrotic stage of TED.

#### 6.3.5 Targeting extracellular matrix remodeling

Targeted inducers of matrix metalloproteinases (MMPs) exert complex effects on macrophage function in thyroid eye disease by regulating extracellular matrix remodeling: on the one hand, they may break the physical confinement of macrophages by moderately degrading excessively deposited fibrotic matrix, thereby promoting a shift from a pro-fibrotic phenotype towards an anti-inflammatory reparative state; conversely, excessive or inappropriate MMP activation may liberate substantial quantities of matrix-sequestered inflammatory cytokines and active fragments, thereby exacerbating local inflammation and recruiting additional macrophages, leading to disease recurrence (175). Therefore, the core of this strategy lies in precisely controlling the timing, intensity, and spatial specificity of MMP activation, thereby remodeling the tissue microenvironment to favor the normalization of macrophage function rather than disrupting its homeostatic equilibrium. This offers a novel approach for indirectly modulating immune cell function by regulating the extracellular physicochemical microenvironment.

### 6.4 Challenges and future directions

In the exploration of treatments for thyroid eye disease, intervention strategies targeting the continuum of macrophage functional states present multiple challenges while simultaneously offering opportunities for breakthrough therapies. Future precision treatment systems will be built upon three pillars: dynamic monitoring, time-sequenced intervention, and model innovation.

First, it is imperative to establish a multidimensional biomarker system capable of real-time tracking of macrophage evolution along the functional continuum. This necessitates moving beyond conventional protein markers (such as sCD163) to integrate single-cell resolution transcriptional regulatory features (STAT/IRF/PPARγ signaling activity) with epigenetic landscapes (such ashistone modifications and chromatin accessibility). This approach enables precise spatio-temporal positioning of macrophage function, thereby providing navigational guidance for personalized therapeutics.

Secondly, therapeutic strategies must align closely with the temporal progression of the functional continuum, progressing towards dynamic precision interventions. In the early stages of disease, macrophage polarization towards the pro-inflammatory state can be suppressed by developing small-molecule inhibitors targeting key pro-inflammatory transcription factors (such as STAT1), or by regulating chromatin accessibility of pro-inflammatory gene clusters using epigenetic editing tools (such as the CRISPR/dCas9 system). Concurrently, interventions targeting their glycolytic metabolic dependency (such as using glycolytic inhibitors) can achieve regulation at the metabolic-epigenetic crossroads. In disease late stages, strategies should pivot towards reversing the pro-fibrotic transformation of macrophages. This encompasses employing histone deacetylase inhibitors or DNA demethylating agents to disrupt their epigenetic consolidation, combined with TGF-β/Smad pathway inhibitors to reverse the fibrotic transcriptional program. Furthermore, development of microenvironment-responsive smart nanodelivery systems may be explored. These systems could selectively release therapeutics to distinct macrophage subpopulations in different functional states based on local inflammatory/fibrotic marker expression, enabling ‘on-demand’ treatment.

Finally, the innovation of research models forms the cornerstone for validating these strategies. It is imperative to construct patient-derived 3D organoid models that not only mimic the pathological microenvironment of human TED but also reproduce the dynamic evolution of the macrophage functional state continuum alongside its underlying transcriptional and epigenetic regulatory networks. This platform will be employed to test novel combination therapies, such as concurrently targeting the JAK/STAT pathway and BET proteins, whilst exploring cutting-edge cellular fate reprogramming techniques—including converting pro-fibrotic macrophages into anti-inflammatory phenotypes. Ultimately, it will provide a translational engine to propel TED treatment from static immunosuppression towards dynamic immune remodeling.

## 7 Conclusion

The pathological progression of thyroid eye disease represents a dynamic process jointly mediated by immune cells and orbital tissue cells. This review systematically elucidates that macrophages, by virtue of their high plasticity along a functional continuum, exert a central regulatory role in the development of thyroid eye disease. They not only execute inflammatory responses but also function as a ‘dynamic regulatory hub’ within the orbital immune microenvironment. Capable of integrating multiple signals—including TSHR/IGF-1R signaling, chemokine networks, metabolic stress, and intercellular interactions—macrophages dynamically modulate their positioning and functional output along the functional continuum through transcriptional reprogramming and epigenetic remodeling. In the early disease phase, macrophages exhibit a pro-inflammatory bias, releasing pro-inflammatory factors such as IL-6 and TNF-α via glycolysis-dependent metabolism and transcriptional networks including NF-κB and STAT1, thereby driving inflammatory cascades. During disease progression, macrophages shift towards a pro-fibrotic state. Their functional reorientation is synergistically driven by transcriptional programs such as TGF-β/Smad and STAT3, alongside specific epigenetic modifications (such ashistone deacetylation, altered DNA methylation patterns). This facilitates pathological fibrosis through TGF-β and PDGF secretion, alongside activation of pathways like GAS6-AXL. Moreover, through intercellular communication and microenvironmental feedback with fibroblasts, adipocytes, and T/B lymphocytes, macrophages collectively sustain the chronicity and recurrence tendency of TED. This explains why anti-inflammatory therapies alone yield limited efficacy in advanced fibrotic stages where epigenetic consolidation and transcriptional program locking have already occurred. Moving forward, dynamic monitoring of the macrophage functional state continuum, coupled with targeted interventions at key transcriptional nodes and epigenetic regulatory mechanisms, holds promise for developing more temporally precise and stratified therapeutic strategies for TED.
